# Marked Decrease Over Time in Conversion Surgery After Active Surveillance of Low-Risk Papillary Thyroid Microcarcinoma

**DOI:** 10.1089/thy.2020.0319

**Published:** 2021-02-12

**Authors:** Takahiro Sasaki, Akira Miyauchi, Yasuhiro Ito, Takumi Kudo, Nobuaki Kanemura, Tsutomu Sano, Shiori Kawano, Masatoshi Yamamoto, Makoto Fujishima, Hiroo Masuoka, Takuya Higashiyama, Minoru Kihara, Akihiro Miya

**Affiliations:** ^1^ Department of Head and Neck Surgery, Kuma Hospital, Kobe, Japan.; ^2^ Department of Surgery, Kuma Hospital, Kobe, Japan.; ^3^ Department of Internal Medicine, Kuma Hospital, Kobe, Japan.

**Keywords:** papillary thyroid microcarcinoma, active surveillance, conversion surgery, lymph node metastasis, tumor size, tumor volume-doubling rate

## Abstract

***Background:*** Active surveillance for low-risk papillary microcarcinoma (PMC) of the thyroid is an accepted and safe management strategy. However, some patients undergo conversion surgery after the initiation of active surveillance for various reasons. We investigated the reasons for conversion surgery and whether and how they changed over time.

***Methods:*** We enrolled 2288 patients with PMC who underwent active surveillance. Of these, 162 (7.1%) underwent conversion surgery >12 months after initiating active surveillance due to disease progression (57 patients), patient preference (43 patients), physician preference (31 patients), other associated thyroid or parathyroid diseases (24 patients), and other reasons (7 patients). We analyzed cumulative conversion rates not only in the whole cohort but also in the first three major subsets based on the reasons for surgery. We also divided our whole cohort into two groups based on the period of active surveillance commencement: the first-half group (February 2005–November 2011; 561 patients) and the second-half group (December 2011–June 2017; 1727 patients).

***Results:*** The criteria for PMC progression did not differ between the first- and second-half groups. The proportion of female patients in the physician preference group was significantly higher than that in the disease progression and the patient preference groups. Tumor size at surgery was larger, and tumor volume-doubling rate was higher in the disease progression group than in the other two groups. Patients in the second-half group were significantly less likely to undergo conversion surgery than those in the first-half group. Furthermore, conversion surgery rates in the second-half group were significantly lower than those in the first-half group in the patient preference, physician preference, and disease progression groups.

***Conclusions:*** Patients with PMC in the second-half group were significantly less likely to undergo conversion surgery than those in the first-half group regardless of the reason. This is probably because data accumulation of favorable outcomes with active surveillance significantly contributed to physicians' confidence and patients' trust and understanding of this disease.

## Introduction

Over the last 30 years, the incidence of thyroid carcinomas has increased, but mortality has remained stable in most studies. The increased incidence is mostly due to the detection of incidental small papillary thyroid carcinomas (PTC) using high-resolution imaging. A large portion of the detected tumors are papillary microcarcinomas (PMCs), measuring only ≤1 cm, and the vast majority of PMCs do not show high-risk features such as significant extrathyroid extension and presence of clinically suspicious lymph nodes or distant metastases (1–3).

In 1993, active surveillance for adult PMC was proposed by Akira Miyauchi and initiated at the Kuma Hospital (Kobe, Japan), while the Cancer Institute Hospital (Tokyo, Japan) started active surveillance in 1995. Given the favorable outcomes of patients who underwent active surveillance in these two Japanese institutions (4–11), this management strategy was adopted in the guidelines issued by the Japanese Association of Endocrine Surgeons (JAES)/Japanese Society of Thyroid Surgery (JSTS) (presently Japan Association of Endocrine Surgery) in 2011 (12) and by the American Thyroid Association (ATA) in 2015 (13).

A subset of patients underwent conversion surgery after the initiation of active surveillance. Our definition for disease progression is (i) tumor enlargement by ≥3 mm or (ii) new appearance of lymph node metastasis. These criteria of disease progression have not changed since active surveillance was implemented. However, in a real-world setting, patients have undergone conversion surgery for various reasons other than disease progression. In this study, we aimed to investigate the trends and the reasons for conversion surgery after the initiation of active surveillance and whether and how they changed over time at Kuma Hospital.

## Materials and Methods

From February 2005, when the electronic medical recording system was introduced at Kuma Hospital, to June 2017, 3769 patients were diagnosed with low-risk PMC using ultrasound-guided fine-needle aspiration (FNA) cytology and high-resolution ultrasound imaging with additional computed tomography scan if required. Of these, 1481 patients (39.3%) preferred to undergo immediate surgery within 12 months after diagnosis, and the remaining 2288 (60.7%) chose active surveillance. Patients who chose active surveillance were followed up regularly with ultrasonography and thyroid function tests once or twice a year.

When we judged disease progression as described above, we considered and reasoned with patients about conversion surgery. However, if tumor enlargement was not associated with lymph node metastasis, patients could continue active surveillance based on their preference until their tumors grew to >13 mm. Of these 2288 patients, 162 (20 male, 142 female; 7.1%) underwent conversion surgery >12 months after the initiation of active surveillance. Their median age at the initiation of active surveillance was 52 years (13–79 years). Our active surveillance protocol is for adult patients with low-risk PMC. However, parents of young patients strongly wanted active surveillance.

As previously described, our definition of PMC progression includes an increase in the maximal tumor diameter of ≥3 mm and the new appearance of lymph node metastasis. In the present study, we grouped patients who had conversion surgery according to the following reasons: (i) disease progression group; (ii) patient preference group, which included patients who for whatever reason decided to stop active surveillance and undergo conversion surgery despite the lack of disease progression; (iii) physician preference group, which included patients who had conversion surgery following physicians recommendation although their tumors did not fulfill disease progression criteria; (iv) patients who underwent surgery due to other associated thyroid or parathyroid diseases; and (v) other reasons.

For this study, we also adopted the tumor volume-doubling rate (TV-DR) to evaluate growth activity (11). Although tumor volume-doubling time (TV-DT) is a validated method to analyze and describe tumor volume kinetics over time, it has two major limitations: First, if some tumors exhibit a decrease in tumor volume, their TV-DTs are expressed as negative values, which causes a discontinuity with patients who show positive values. Second, the magnitude of the TV-DT values is opposite to that of tumor growth or shrinkage (11). TV-DR, that is, the inverse of TV-DT, can resolve these limitations (11). For calculating TV, measurements of the maximum diameter (D_1_) and the diameter in the direction perpendicular to the maximum diameter (D_2_) of tumors were used. Tumor depth was often unreliable because of ultrasound shadowing. TV was calculated using the ellipsoid equation (π/6 × D_1_ × D_2_^2^). TV-DT was then calculated by serial measurements in each patient as described previously (14). In this study, we adopted TV-DR, the inverse of TV-DT. For these calculations, one can download “Doubling Time, Doubling Rate & Progression Calculator, Ver. 2” at the website of Kuma Hospital (www.kuma-h.or.jp/english).

For evaluating lymph nodes suspected of metastasis, we measured three dimensions (major axis, minor axis, and depth). For the diagnosis of PTC metastasis, we usually performed FNA for suspicious nodes and also measured thyroglobulin levels in the washout of the needles used for FNA.

Chi-square and Fisher's exact tests were used for comparing variables. For the time-sequence analysis, we used the Kaplan–Meier method with the log-rank test. All statistical analyses were performed with Stat Flex (View Flex, Tokyo, Japan). *p*-Values <0.05 were considered significant.

All patients with low-risk PMC gave informed decisions for their management strategy; immediate surgery or active surveillance and, if required, conversion surgery. The Ethics Committee at Kuma Hospital waived obtaining informed consent of the patients because of the retrospective nature of the present study.

## Results

The reasons for undergoing conversion surgery were as follows: disease progression (57 patients), patient preference (43 patients), physician preference (31 patients), enlargement of coexisting benign nodules (21 patients), onset of primary hyperparathyroidism (3 patients), initiation of immunosuppressive therapy for autoimmune diseases such as rheumatoid arthritis (2 patients), and unknown (5 patients). Physicians planning immunosuppressive therapies often requested us to resect the PMC to preclude possible adverse effects. The disease progression group included 42 patients with tumor enlargement and 15 patients with new lymph node metastasis; none of the patients had both features. For physician preference, multiple endocrine surgeons, endocrinologists, and head and neck surgeons saw the patients for a long period of time at the Kuma Hospital. Some physicians tended to recommend surgery for minor findings such as a slight increase in tumor size, tumor location near the trachea or dorsal portion of the thyroid lobe, or possible presence of multiple foci.

First, we analyzed differences in clinicopathological characteristics among the three major groups (patient preference, disease progression, and physician preference). Table 1 summarizes the clinical characteristics of the three groups of patients who underwent conversion surgery. The proportion of women was significantly higher in the physician preference group than in the other two groups. As expected, tumor size at surgery was larger, and TV-DR was higher in the disease progression group than in the other two groups. Interestingly, 27.9% of patients in the patient preference group and 16.1% of those in the physician preference group had a TV-DR < −0.1/year. Among them, 11.6% in the patient preference group and 6.5% in the physician preference group showed a 50% decrease in their tumor volume, indicating that their tumors shrunk before conversion surgery. In the disease progression group, 5.3% of patients showed tumor shrinkage (TV-DR < −0.1/year), and these patients underwent surgery because of new appearances of lymph node metastasis. None of the other characteristics significantly differed among the three groups. Table 2 compares clinical features between patients with tumor enlargement (*n* = 42) and those with the appearance of nodal metastasis (*n* = 15) in the disease progression group. None of the patients showed both tumor enlargement ≥3 mm and the appearance of nodal metastasis. The TV-DR of a portion of patients showing an appearance of nodal metastasis was negative, indicating that these primary lesions shrunk despite the development of nodal metastasis. However, none of the patients in the disease progression group had a 50% decrease in tumor volume. Age at diagnosis was significantly younger in patients with an appearance of nodal metastasis than in those with tumor enlargement (median age: 40 vs. 56 years, *p* = 0.033). Tumor size at surgery was larger, and TV-DR was higher in patients with tumor enlargement than in those with lymph node metastasis (median: 12.5 vs. 7.0 mm, *p* < 0.001; 0.62 vs. 0.16/year, *p* = 0.006, respectively).

**Table 1.** Clinical Characteristics of the Three Groups of Patients Who Underwent Conversion Surgery

	Patient preference group (*n* = 43)	Disease progression group (*n* = 57)	Physician preference group (*n* = 31)	Significance
Male/female (percentage of female)	7/36 (83.7%)	8/49 (86.0%)	0/31 (100.0%)	*p* = 0.018 (^*^) *p* = 0.015 (^**^)
Age at diagnosis (years)^a^	51 (20–72)	50 (13–79)	51 (21–74)	n.s.
Age at surgery (years)^a^	53 (22–77)	53 (15–83)	54 (24–76)	n.s.
Period from diagnosis to surgery (years)^a^	2.2 (1.2–7.8)	2.9 (1.0–8.8)	2.8 (1.1–8.1)	n.s.
Study period (years)^a^	8.9 (2.7–13.7)	8.0 (2.6–13.3)	8.9 (2.4–14.0)	n.s.
Family history of thyroid carcinoma: yes	2 (4.7%)	3 (5.3%)	1 (3.2%)	n.s.
TgAb(+)	11 (25.6%)	20 (35.1%)	10 (32.3%)	n.s.
Tumor size (mm)				
At diagnosis^a^	8.0 (5–10)	7.0 (3–10)	8.0 (3–10)	n.s.
At surgery^a^	8.0 (5–12)	12.0 (4–18)	9.0 (4–12)	*p* = 0.014 (^*^) *p* < 0.001 (^**^)
TV-DR (/year)^a^	0.10 (−1.02–0.80)	0.53 (−0.67–2.87)	0.10 (−1.02–0.80)	*p* < 0.001 (^*^) *p* < 0.001 (^**^)

^a^ Median value (range).

n.s., not significant; TgAb, thyroglobulin antibodies; TV-DR, tumor volume doubling rate.

**Table 2.** Clinical Features Between Patients with Tumor Enlargement and Those with Appearance of Nodal Metastasis in the Disease Progression Group

	Tumor size increase of ≥3 mm (*n* = 42)	Appearance of lymph node metastasis (*n* = 15)	Significance
Male/female (percentage of female)	5/37 (88.1%)	3/12 (80.0%)	n.s.
Age at diagnosis (years)^a^	56 (13–79)	40 (29–65)	*p* = 0.033
Age at surgery (years)^a^	59.5 (15–83)	43 (33–69)	n.s. (*p* = 0.063)
Period from diagnosis to surgery (years)^a^	3.0 (1.0–8.8)	2.8 (1.3–4.2)	n.s.
Study period (years)^a^	8.0 (2.6–13.2)	9.6 (5.2–13.3)	n.s.
Family history of thyroid carcinoma: yes	1 (2.4%)	2 (13.3%)	n.s.
TgAb(+)	13 (31.0%)	7 (46.7%)	n.s.
Tumor size(mm)			
At diagnosis^a^	8.0 (3–10)	7.0 (4–10)	n.s.
At surgery^a^	12.5 (7–18)	7.0 (4–12)	*p* < 0.001
TV-DR (/year)^a^	0.62 (0.09–2.98)	0.16 (−0.67–2.87)	*p* = 0.006

^a^ Median value (range).

**FIG. 1.**
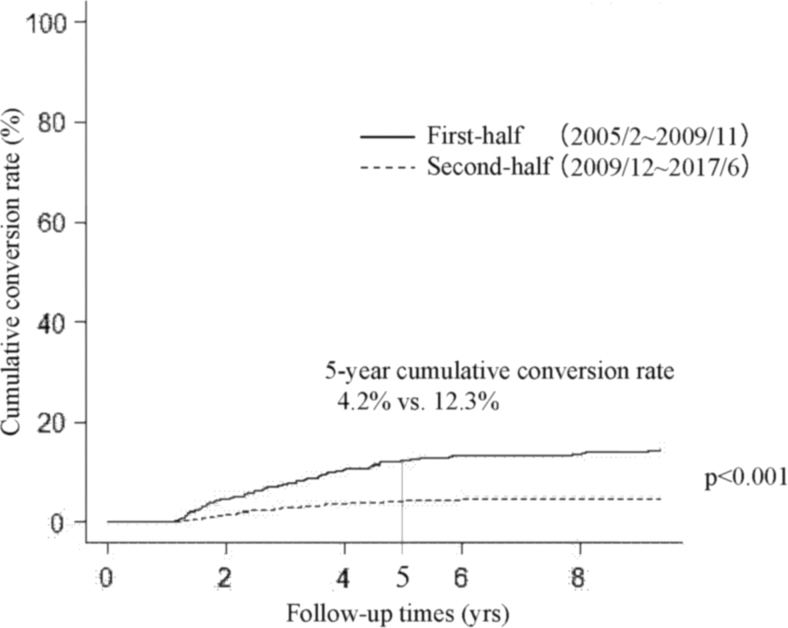
Kaplan–Meier curves of cumulative conversion rates in the whole sample for the first-half and second-half groups.

**FIG. 2.**
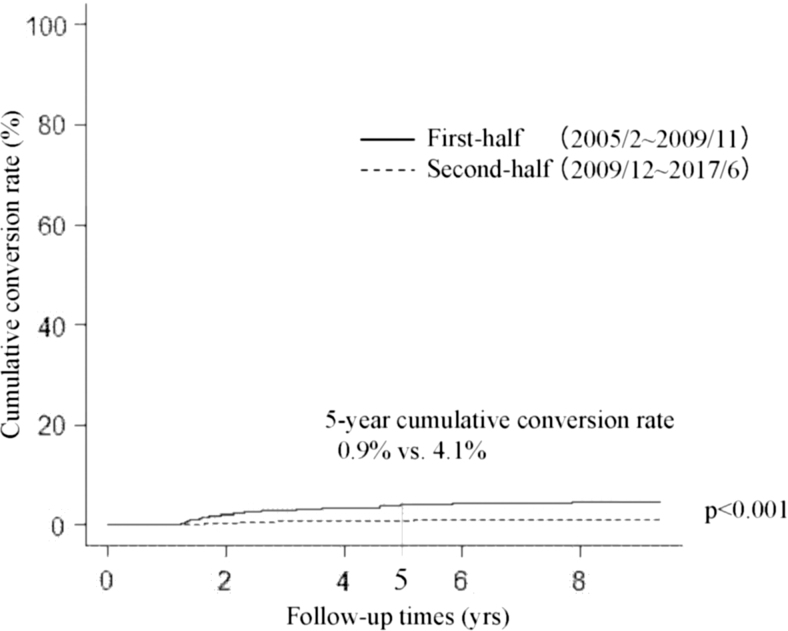
Kaplan–Meier curves of cumulative conversion rates in the patient preference group of patients with papillary microcarcinoma in the first-half and second-half periods.

**FIG. 3.**
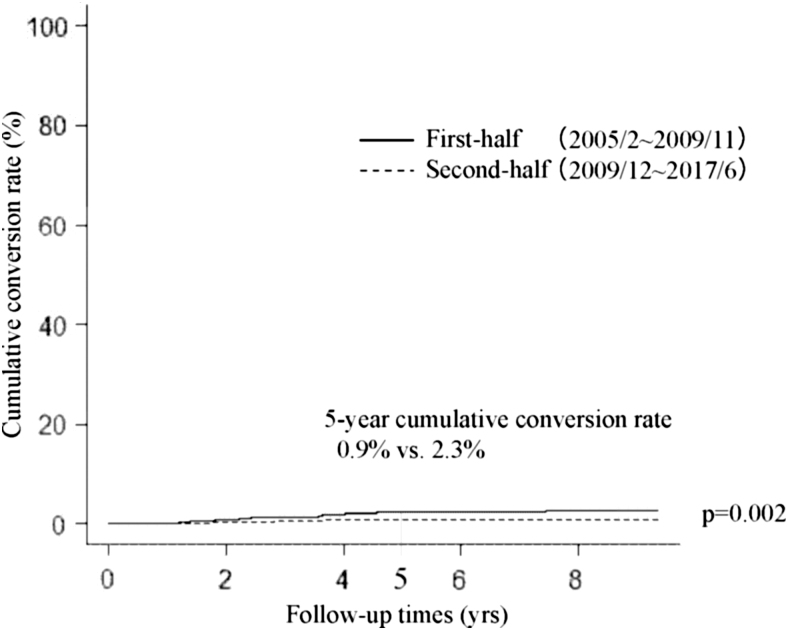
Kaplan–Meier curves of cumulative conversion rates in the physician preference group of patients with papillary microcarcinoma in the first-half and second-half periods.

**FIG. 4.**
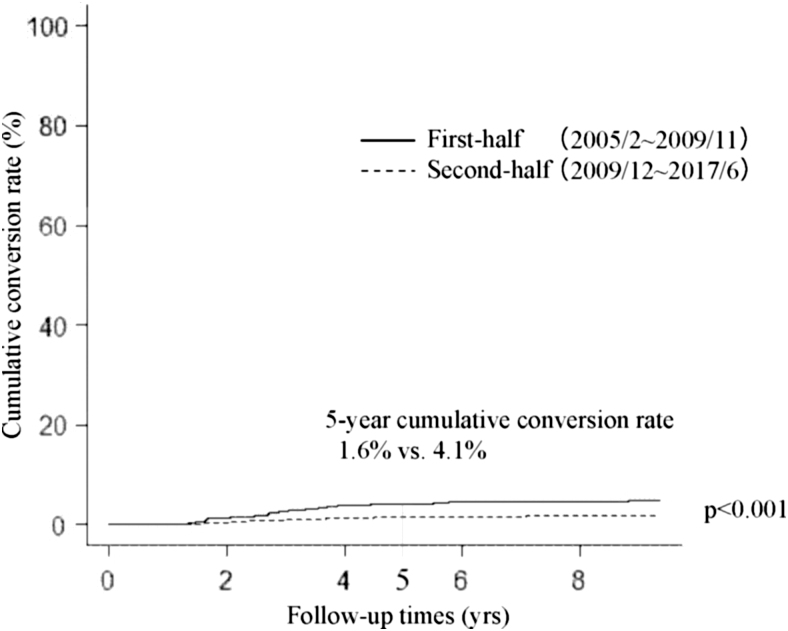
Kaplan–Meier curves of cumulative conversion rates in the disease progression group of patients with papillary microcarcinoma in the first-half and second-half periods.

We next divided our whole cohort (2288 patients) into two groups, the first-half group (from February 2005 to November 2011, 561 patients) and the second-half group (from December 2011 to June 2017, 1727 patients) based on the start of active surveillance. Incidentally, both groups included the same number of patients who underwent conversion surgery (81 patients each). Figure 1 compares the Kaplan–Meier curves of cumulative conversion surgery rates of patients in the first-half and second-half groups. Patients in the second-half group were significantly less likely to undergo conversion surgery than those in the first-half group (*p* < 0.001); the 5-year cumulative conversion rates of the second-half and first-half groups were 4.2% and 12.3%, respectively. Furthermore, cumulative conversion rates in the second-half group were significantly lower than those in the first-half group for patient preference (*p* < 0.001, 5-year cumulative conversion rate 0.9% vs. 4.1%) (Fig. 2), physician preference (*p* = 0.002, 5-year conversion surgery rate 0.9% vs. 2.3%, Fig. 3), and disease progression subgroups (*p* < 0.001, 5-year conversion surgery rate 1.6% vs. 4.1%, Fig. 4).

Table 3 shows the comparison of clinical features of patients with PMC showing enlargement ≥3 mm between the first-half and second-half groups. Except for the study period, none of these factors significantly differed between the two groups. Particularly, tumor size at conversion surgery did not differ between the two groups. In the subset of patients with PMC and an appearance of lymph node metastasis, the study period was longer, and the metastatic node depth was shorter in patients in the first-half group than those in the second-half group (Table 4).

**Table 3.** Clinical Features of Patients with Papillary Microcarcinoma Showing Enlargement ≥3 mm Between the First-Half and Second-Half Groups

	First-half group (*n* = 18)	Second-half group (*n* = 24)	Significance
Male/female (percentage of female)	1/17 (5.6%)	4/20 (16.7%)	n.s.
Age at diagnosis (years)^a^	51.5 (27–76)	58 (13–79)	n.s.
Age at surgery (years)^a^	54 (28–80)	61 (15–83)	n.s.
Period from diagnosis to surgery (years)^a^	3.2 (1.4–8.8)	2.7 (1.0–7.1)	n.s.
Study period (years)^a^	9.8 (5.1–13.2)	5.9 (2.6–9.1)	*p* < 0.001
Tumor size (mm)			
At diagnosis^a^	8.0 (5–9)	7.0 (4–10)	n.s.
At surgery^a^	12.0 (8–17)	13.0 (7–18)	n.s.
Difference	4.0 (3–8)	5.0 (3–9)	n.s.
TSH suppression (LT4 intake before operation)	4 (22.2%)	5 (20.8%)	n.s.

^a^ Median value (range).

LT4, levothyroxine; TSH, thyrotropin.

**Table 4.** Clinical Features of Patients with Appearance of Nodal Metastasis Between the First-Half and Second-Half Groups

	First-half group (*n* = 9)	Second-half group (*n* = 6)	Significance
Male/female (percentage of female)	1/8 (11.1%)	2/4 (33.3%)	n.s.
Age at diagnosis (years)^a^	48 (29–65)	37.5 (30–56)	n.s.
Age at surgery (years)^a^	50 (33–69)	40 (33–57)	n.s.
Period from diagnosis to surgery(years)^a^	2.7 (1.3–4.2)	2.9 (1.3–3.7)	n.s.
Study period (years)^a^	10.5 (5.8–13.3)	6.4 (5.2–7.8)	*p* < 0.01
Lymph node size (mm)			
Major axis	7.0 (4–26)	13.5 (8–24)	n.s.
Minor axis	4.0 (3–7)	5.5 (4–10)	n.s.
Depth	5.0 (2–7)	9.5 (5–10)	*p* < 0.05
TSH suppression (LT4 intake before operation)	1 (11.1%)	0 (0%)	n.s.

^a^ Median value (range).

## Discussion

This study demonstrated that (i) 7.1% (162 of 2288) of patients underwent conversion surgery after the initiation of active surveillance for various reasons; (ii) of these, only 57 patients (35.2%) showed disease progression; and (iii) dividing patients into the first-half and second-half groups based on the period of active surveillance commencement showed that the cumulative conversion rate in the second-half group was significantly lower than that in the first-half group.

Disease progression was defined as tumor enlargement by ≥3 mm or new appearance of lymph node metastases at the implementation of this management strategy in 1993. When we judged that disease had progressed, we considered and reasoned with patients about conversion surgery. However, over time, with the accumulation of favorable outcomes with active surveillance, we gradually shifted to continue active surveillance for slightly enlarged tumors without lymph node metastasis according to patients' preference, until their tumors grow >13 mm. However, in reality, a considerably large number of patients underwent conversion surgery for other reasons. Indeed, in our series, 43 and 31 patients underwent conversion surgery due to patient preference and physician preference, respectively. As shown in Table 1, tumor size at surgery was smaller and TV-DR was lower in these two groups than in the disease progression group.

The disease progression group consists of two subcategories; PMCs with an increase in size of ≥3 mm and those with new lymph node metastasis. Tumor size was smaller, and TV-DR was lower in the PMC group with the appearance of lymph node metastasis than in the enlarged PMC group, indicating that the biological significance of tumor enlargement and lymph node metastasis differs. Interestingly, none of the present patients had both features. A previous study also reported that the coexistence of these events is rare (15). The present report showed that 20% of patients showing a new appearance of lymph node metastasis had a TV-DR < −0.1/year, indicating that the development of lymph node metastasis can occur even though primary lesions are stable or even shrink.

We investigated differences in the cumulative conversion rate of patients in the first-half and second-half groups (before and after December 2011). Interestingly, cumulative conversion rates of patients in the second-half group were significantly lower than those of patients in the first-half group not only in the whole group, but also in the subsets of patient preference, physician preference, and disease progression groups. It was previously demonstrated that the proportion of patients with low-risk PMC who chose active surveillance has gradually increased from 42% between 2003 and 2006 to 64% between 2007 and 2013, and 88% since 2014 (16). This may be because the accumulation of favorable outcomes for active surveillance made attending physicians explain its merits to their patients based on more convincing data. Also, this may have resulted in patients' trust in their physicians and active surveillance management of their disease. This can explain the significantly lower cumulative conversion rates during the second-half period in the patient preference and physician preference groups. The cumulative conversion rate in the second-half period was also significantly lower than that in the first-half period in the disease progression group. As shown in Table 3, the median tumor diameter at surgery in the second-half group was 1 mm larger than in the first-half group. Also, the difference in size at surgery and the start of active surveillance was 1 mm larger in the second-half group than in the first-half group. These data reflect our gradual shift to continuing active surveillance for slightly larger tumors without lymph node metastasis based on patients' preference.

Recently, many patients have chosen not to undergo immediate conversion surgery when their PMCs enlarged by 3 mm, a preference that has been generally accepted by attending physicians, who continue active surveillance until the maximum size of the tumors reaches around 13 mm or there is a possibility of the invasion of nearby structures such as the recurrent laryngeal nerve and the trachea. This modification in the active surveillance management seems to be appropriate since the growth activity of PMCs often significantly decreases after tumors grow ≥3 mm (17). Only a portion of PMCs show further growth; conversion surgery should be done for these cases. This may explain, at least in part, the lower cumulative conversion rate during the second-half period than that during the first-half period in the disease progression group.

In patients with PMC showing node metastasis, lymph node depth was higher in the second-half group that in the first-half group. The shape of a normal or reactive neck lymph node is like a deflated rugby ball, being longest in the craniocaudal direction. We think that depth, the smallest of the three dimensions of a lymph node, is the most important when evaluating for PMC metastasis to lymph nodes. A long lymph node depth is one of the ultrasound features of metastatic disease. The difference found may be because FNA for suspicious nodes was performed at an earlier stage in the first-half group, whereas in the second-half group, physicians tended to continue active surveillance without FNA until the metastatic node findings became more apparent.

This study has some limitations. This was a single-center retrospective study. Many attending physicians participated in the active surveillance of PMC; the threshold of recommending conversion surgery might thus vary from physician to physician and over time as well. At the diagnosis and evaluation of the disease, patients were offered immediate surgery and active surveillance, and patients chose one of them. Hence, this was not a randomized study. Some patients who chose active surveillance might have changed their mind to undergo surgery within one year. These patients were included in the immediate surgery group in the present study. The explanations by physicians might have contained some differences in details, resulting in varying levels of conviction about the management over time; this was seen in our previous study on the trends in the implementation of active surveillance at Kuma Hospital (16).

In conclusion, regardless of the reasons, patients with PMC in the latter part of our experience were significantly less likely to undergo conversion surgery than those in the earlier experience with active surveillance. This is probably because the accumulation of favorable outcomes with this management strategy significantly contributed to physicians' confidence and patients' trust and understanding of the disease. We hope that our experience can be useful for institutions that have begun and are trying to begin active surveillance for PMC.
